# Education and Provision of a Pest Management Tool Kit to Residents in Low-Income Housing: Effect on Cockroach Reduction

**DOI:** 10.3390/insects17050483

**Published:** 2026-05-09

**Authors:** Xiaodan Pan, Souvic Sarker, Jin-Jia Yu, Richard Cooper, Changlu Wang

**Affiliations:** Department of Entomology, Rutgers University, New Brunswick, NJ 08901, USA

**Keywords:** IPM education, pest control, multi-unit dwellings, cockroaches

## Abstract

High rates of pest infestations are frequently reported in low-income homes in the U.S. However, even with access to effective insecticides, most residents are unaware of how to properly control indoor pests. We designed and evaluated the effect of in-home integrated pest management (IPM) education and provision of pest management tool kit on German cockroach (*Blattella germanica* L.) infestations. Twenty-nine apartments were enrolled based on the presence of German cockroaches. They were divided into two groups: an intervention group (12) and a control group (17). The intervention group received a cockroach prevention and control flyer designed by the researchers, gel bait, a bottle of boric acid dust, sticky traps, and in-person brief education on how to prevent and control cockroaches. The control group did not receive education or pest control materials. The intervention resulted in a significantly higher reduction in cockroach numbers and infestation rates compared to the control. At 6 months, the geometric mean cockroach count per apartment decreased by 99% in the intervention group and 51% in the control group. Cockroaches were no longer detected in 58% and 6% of the intervention and control groups, respectively. However, the IPM education did not result in significant improvement in the sanitation rating.

## Simple Summary

German cockroaches are the most common pest in low-income housing across the U.S. Many residents are unaware of effective ways to control them. This study tested whether educating residents about integrated pest management (IPM) and providing cockroach control products could help reduce cockroach infestations. A total of 29 apartments with cockroach infestations in New Brunswick, NJ, USA, were enrolled in a study. Among them, 12 apartments received gel baits, a bottle of boric acid dust, sticky traps, a flyer on cockroach management, and in-person brief education (“intervention” group), while the other 17 apartments did not receive any of these items (“control” group). Even though the IPM education did not result in significant improvement in sanitation ratings over a six-month period, the geometric mean German cockroach count per apartment decreased by 99% in the intervention group and 51% in the control group. At 6 months, cockroaches were no longer detected in 58% of the intervention group and 6% of the control group. These findings demonstrate that brief in-person IPM education, combined with the provision of effective control materials, reduces German cockroach infestations in low-income communities without direct involvement of housing or pest control staff.

## 1. Introduction

Persistent and high prevalence of indoor pests is often reported in low-income communities in the U.S. [1,2,3,4]. The three most common indoor pests are German cockroaches (*Blattella germanica* L.), house mice (*Mus musculus domesticus* (Schwarz and Schwarz)), and bed bugs (*Cimex lectularius* L.). A survey conducted in four cities in New Jersey from 2018 to 2019 found that 56% of low-income apartments had at least one of these pests [3]. All are important pests that affect people’s health or quality of life. The health risks include disease transmission, food contamination, production of allergens, bites, psychological distress, etc. [5,6,7,8]. Besides these major pests of public health importance, many other pests occur in homes [9,10,11]. Most are considered nuisance pests; however, some cause damage to stored products, fabrics, structures, and other items [9].

Abbar et al. (2022) [3] reported that cockroaches were present in 37% of the 3008 inspected low-income apartments in four cities in NJ, USA. German cockroaches were the most abundant species, accounting for 97.8% of the cockroaches in roach-infested apartments [3]. The prevalence of cockroaches largely stems from their adaptive behavior and high reproductive rate. German cockroach infestations are mostly confined to kitchens and bathrooms, where food and water are always available; however, they will readily infest entire apartments. German cockroaches feed on human food, pet food, and other organic materials present in homes. Food debris, unwashed dishes, open food containers, and dirty ovens provide cockroaches with a steady and reliable source of food. The proximity and connectivity between apartments allow cockroaches to spread from one unit to another and lead to chronic infestations [12]. Cockroach skins, feces, and dead bodies contain allergens that trigger asthma or allergic reactions [13]. Among the pests in homes, cockroaches represent the most common cause of airway allergic sensitization and bronchial asthma worldwide. There are at least 11 allergens have been identified from the German cockroach, with Bla g 1 and Bla g 2 known to be asthma triggers [14].

Several factors contribute to the high prevalence of cockroach infestations in low-income communities, including the presence of homes with poor sanitation conditions in a building, lack of awareness of the presence of cockroaches among residents, and people’s attitudes toward cockroach infestations [15,16], and ineffective self-control by residents. Pest control services offered by housing authorities are typically low-bid and often fail to provide adequate pest control results [17]. This type of service is complaint-based; if no complaint is submitted, the apartment is not serviced during the monthly visits. When servicing an infested apartment, technicians often rely solely on baits or sprays without conducting post-treatment monitoring of the results. The service time is extremely short (less than a minute), and the amount of material (less than a gram per apartment) is insufficient [17]. A high percentage of residents attempt to control cockroaches themselves due to convenience, privacy, low cost, or dissatisfaction with the pest control services offered by the building management. A survey of 384 residents found that 74% implemented methods themselves to control cockroaches [15]. Consumers have access to many products, including professional ones. Over-the-counter consumer insecticide sprays (liquid or aerosol) were the most commonly used type of product, but their effectiveness is questionable [18]. Also, residents may fail to use effective products correctly. Moreover, the frequent use of insecticide sprays leads to contamination of the indoor environment [19].

Application of bait formulations is the most popular and effective method for controlling German cockroaches [20]. It causes less contamination to the indoor environment than sprays. Gel bait-based cockroach management programs were highly successful in controlling German cockroaches in field studies [4,19,21,22]. Many effective bait products containing abamectin, dinotefuran, fipronil, indoxacarb, etc., are available through distributors or online vendors. However, fewer bait products are available to consumers in retail stores in the USA. In addition, Chungsawat et al. (2025) found that all tested over-the-counter bait products were ineffective against field-collected German cockroaches [23]. Similarly, a separate study found that two commonly used consumer bait products containing fipronil are less effective than a combination of three professional products [24]. Lack of readily accessible, effective gel bait products and public knowledge of effective control materials hinder the effectiveness of do-it-yourself cockroach control efforts.

Given the prevalence of cockroaches in housing communities and use of ineffective materials by residents, educating clients on cockroach integrated pest management (IPM) including proper sanitation, clutter reduction, application of a combination of non-chemical and effective chemical tools, and follow-up monitoring to eliminate cockroaches, is urgently needed [25]. University extension services and government agencies regularly provide guidance and educational materials to the public about indoor pest IPM. One prominent source of these resources is “Stop Pests in Housing” (https://www.stoppests.org/what-is-ipm/guide/, accessed on 27 April 2026). This website contains the most comprehensive educational materials on IPM in affordable housing. The Stop Pests in Housing program and university extension personnel also host educational seminars for policy makers, housing staff, and the public on pest prevention and control. However, data on the effectiveness of these educational approaches in reducing pests is very limited. There is scarce information for consumers on which bait products to choose. McConnell et al. (2005) evaluated the effect of in-home education and the provision of a bottle of boric acid and caulking materials on cockroach numbers [26]. The geometric mean cockroach number in the intervention homes at the 4-month follow-up visit was 60% lower than in the non-intervention homes. But the infestation rate in the intervention group remained similar (from 59% at 0 months to 57% after 4 months). A study evaluated the effect of health education, presented in the form of slide presentations and printed materials, on cockroach infestations in Seoul, Korea. The cockroach infestation rates decreased from 62% to 23% after two years [27]. Similarly, Crider (2010) found education of elderly residents and staff caused lower cockroach counts after 5 months [28]. However, both studies lacked control groups. There is a need to develop a proven, effective IPM education strategy, which is highly important for future educational efforts. In this study, we designed and tested a program that included brief in-home education and provision of effective cockroach control materials and compared it with a control group. This study differs from previous studies in that we provided proven-effective cockroach management materials and included a control group. Here, we report findings on the effect of resident IPM training plus provision of cockroach control materials on cockroach infestations in low-income apartment buildings.

## 2. Materials and Methods

### 2.1. Study Site and Selection of Apartments

**Figure 1:**
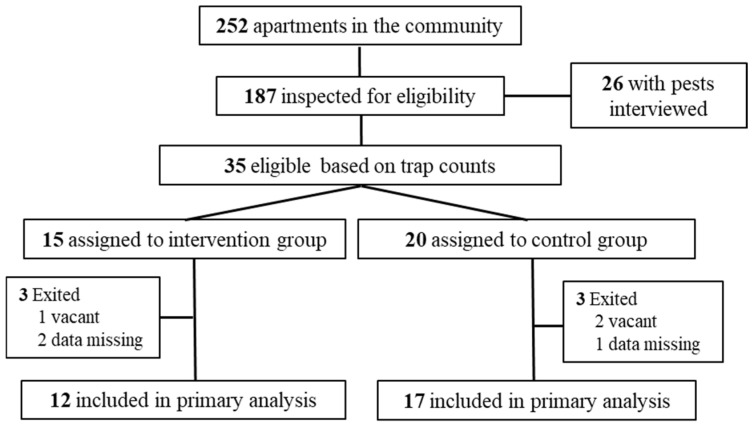
Diagram showing the study design. More apartments were assigned to the control group because the tenants were not at home during our visits.

The study was conducted in a low-income community in New Brunswick, NJ, USA. The community contained 252 apartments in 38 buildings. Each building contained 4 or 8 apartments. Each apartment had 1 or 2 bedrooms. A pest control provider hired by the housing authority visited the community monthly and applied gel bait for cockroaches based on access and complaints received. However, very little bait (<1 g) was used per apartment for cockroach control based on observations from the researchers and the management staff. To select eligible apartments for the study, we placed four Trapper insect monitors (1/3 of the whole trap) (Bell Laboratories Inc., Madison, WI, USA) per apartment in 187 apartments and retrieved them after 7 days (Figure 1). The remaining apartments in the community were not accessed. The trap locations were inside the kitchen cabinet under the sink, beside the stove, beside the refrigerator, and beside the toilet.

If residents were at home during the initial inspection, they were interviewed about pest sightings (such as cockroaches, mice, and bed bugs) and self-control methods (including insecticides, traps, or other methods) (Supplementary Materials S1). We also asked if they were satisfied with the pest control service offered by the building management. A total of 26 residents who had noticed the presence of pests (cockroaches, mice, bed bugs) completed the initial questionnaire. The initial interview was conducted in May 2023. 

Among the 187 apartments that were monitored with sticky traps, 35 apartments (including 24 of the 26 interviewed) had cockroaches captured over a one-week period (Figure 1). Among the 35 apartments, six were excluded from the analysis due to vacancy or data issues during the study (Figure 1). These apartments were divided into two groups. The intervention group included 12 apartments and received IPM education and cockroach control materials. The control group included 17 apartments and did not receive education or control materials. More apartments were assigned to the control group because the tenants were not at home during our visits and thus could not receive in-person education. The sanitation conditions in each apartment were scored following a previous rating system: 1-good, 2-average, 3-poor, or 4-very poor [15]. The “Very poor” home had excessive garbage, leftover food, and food residues on floors and kitchen counters. The clutter conditions were rated as few, moderate, cluttered, or severely cluttered. The two groups had similar mean numbers of cockroaches and housekeeping conditions at the baseline or at the beginning of the study.

### 2.2. Resident Education on Cockroach IPM and Provision of Cockroach Control Materials

**Figure 2:**
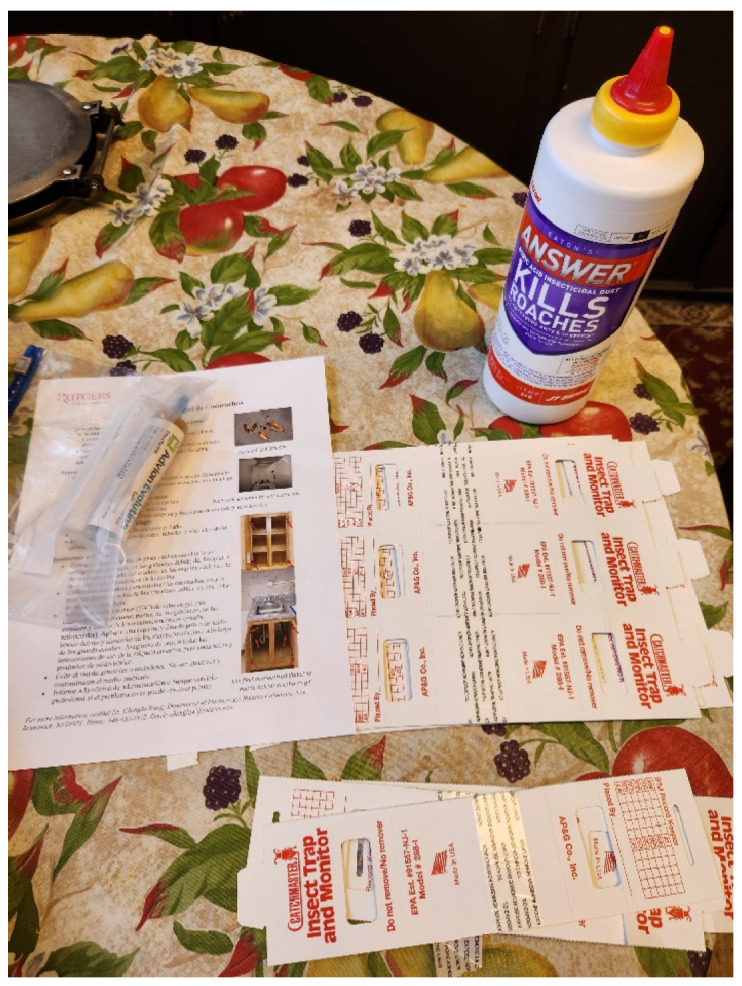
Cockroach control materials provided to a resident.

Within a week of the initial inspection, residents in the intervention group received a one-page brochure on cockroach prevention and control (Supplemental Materials S2). They were also provided with cockroach control materials and shown how to apply them. These materials included five sticky trap insect monitors (each can be split into three pieces) (Catchmaster 100i Insect Monitor, AP&G, Bayonne, NY, USA), a bottle of boric acid dust (Answer boric acid dust, J.T. Eaton Co., Twinsburg, OH, USA), a tube of Advion cockroach gel bait (0.06% indoxacarb, Syngenta, Greensboro, NC, USA), and product instructions (Figure 2). These insecticides have been shown to be very effective for controlling cockroaches in a previous study [19]. We did not offer over-the-counter bait products from the retail stores due to their low efficacy [23]. The pest control materials were purchased from a distributor. Researchers provided instructions on how to properly apply the products, and encouraged residents to read the brochure, keep the apartment clean, remove clutter in the kitchen and bathroom, and use the products to monitor and control cockroaches. The goal was to teach residents the importance of proper sanitation and to provide them with the knowledge and skills to properly use effective management tools and avoid using ineffective products (sprays, insect foggers, electronic repellers). The contracted pest control company continued to service the whole community (including the study apartments) monthly based on residents’ complaints during the study period. The contractor was not notified of the study. However, only those who complained and were accessible on the monthly service date were treated, and, as indicated earlier, the existing contractor used a very small amount of bait (1–2 tubes for the whole community).

### 2.3. Follow-Up Inspections and Evaluation of the Effectiveness of Education and Provision of Cockroach Control Materials in Reducing Cockroach Populations

One month and six months later, four Trapper insect monitors were placed in each apartment for a week to monitor the presence of cockroaches. The levels of sanitation (but not clutter) of the apartments were rated. If residents were at home, they were asked the same questions as those at month 0. In addition, researchers asked residents what they had done to control pests in the past 1 month. Residents in four apartments in the intervention group received additional pest control materials (traps, dust, or bait). A different bait, Maxforce FC Select (0.01% fipronil, Envu, Cary, NC, USA), was offered to two of these four apartments to avoid resistance development.

### 2.4. Statistical Analysis

Two-sample *t*-test was used to compare initial sanitation ratings in the intervention and control groups. The mean change in sanitation rating from 0 to 6 months was analyzed using analysis of variance (ANOVA). ANOVA was also used to analyze the logarithmically transformed total trap count per apartment at 0 months and 1 month. The 6-month trap count data were not normally distributed even after transformation and were analyzed using the Wilcoxon Two-Sample Test. The percentages of cockroach count change from month 0 to month 1 and month 6 were calculated and subjected to the Wilcoxon Two-Sample Test to determine whether there is a significant difference between the intervention and control groups. Chi-square test was used to compare the percentage of apartments with zero cockroach counts at month 6. All analyses were performed using SAS software version 9.4 [29].

## 3. Results

### 3.1. Community Characteristics and Home Assessment of the Surveyed Residents

Based on the 26 residents who initially reported the presence of pests in their homes, the characteristics of the residents and their home environments are summarized in Table 1. Among them, 4 (15%) apartments had both German cockroaches and house mice. One apartment (4%) had both bed bugs and house mice. Eighty-five percent of residents tried to control pests themselves. The three most used methods were insecticide spray (26%), sticky traps (24%), and baits (15%). The other tools and methods included snap traps, electronic repellers, plugging holes, cleaning, essential oils, insect bombs, and insecticide dust. Despite the presence of pests, 65% of residents were satisfied with the pest control service provided by the housing authority.

Eight residents from the intervention group apartments filled out the questionnaire at all three study points. All of them said they used the cockroach control materials we provided, indicating that the in-house IPM education and provision of materials are well accepted by residents. Four of the residents had used insecticide sprays for cockroach control prior to the study. They did not use them during the study period.

### 3.2. Changes in Sanitation Ratings

At the beginning of the study, the mean (±SE) sanitation rating in the intervention group (*n* = 12) and the control group (*n* = 17) were 1.7 ± 0.2 and 2.0 ± 0.1, respectively. They are not significantly different (*t* = 1.41, df = 27, *p* = 0.171). From 0 to 6 months, the mean change in sanitation rating was 0.3 ± 0.7 in the intervention group and 0.1 ± 0.2 in the control group. They are not significantly different between the two groups (*F* = 0.75; df = 1, 23; *p* = 0.397). Therefore, IPM education did not lead to an improvement in sanitation ratings.

### 3.3. Dynamics of Cockroach Counts in the Study Apartments

**Figure 3:**
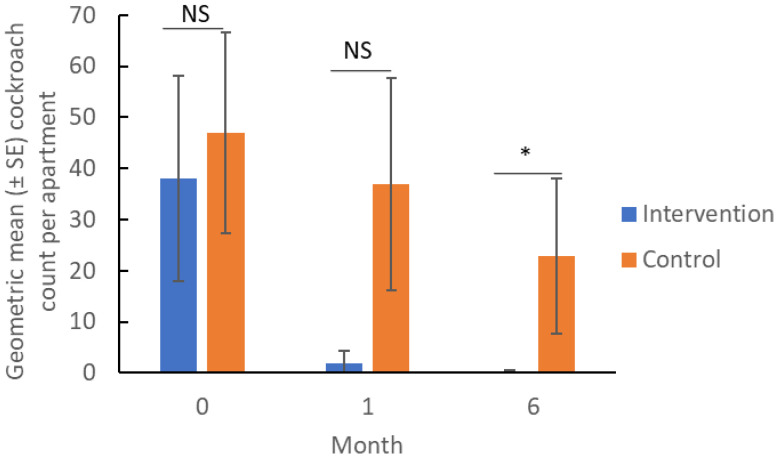
Cockroach counts per apartment based on sticky traps (7-day trapping period) before and after IPM education and provision of cockroach control materials. “NS” indicates no significant difference (ANOVA, *p* > 0.05). Asterisk (*) indicates significant difference (Wilcoxon Two-sample Test, *p* = 0.005).

The only cockroach species found on sticky traps was the German cockroach. At the beginning and 1 month of the study, the geometric mean trap count was not significantly different between the two groups (ANOVA, *p* > 0.05) (Figure 3). The geometric mean counts in the two groups became significantly different after 6 months (*Z* = −2.84, *p* = 0.005). After 1 month, the mean (±SE) percent reduction in counts was 4 ± 55% among the intervention units. In contrast, the count increased by 18 ± 40% among the control units. Although the geometric mean trap counts of the two groups appear very different after 1 month, the percentage change is not significantly different between these groups (Wilcoxon Two-Sample Test, Z = 1.48, *p* = 0.137). Seven apartments (5 in the control group, 2 in the intervention group) had missing data at 1 month, which reduced the power to detect differences between groups. After 6 months, the mean percent reduction in the intervention group was 56 ± 35%; whereas in the control group, it increased by 298 ± 193%. They are significantly different (Wilcoxon Two-Sample Test, *Z* = 2.82, df = 1, *p* = 0.005). The large increase in the control is due to the trap count in one apartment increasing from 4 to 129. The geometric mean cockroach count per apartment decreased by 99% in the intervention group and 51% in the control group. In the intervention group, a significantly higher percentage (58%) of apartments had zero trap catch than that in the control (6%) after 6 months (*χ*^2^ = 9.69, *p* = 0.002). Thus, IPM education, along with the provision of cockroach control materials, is effective in reducing cockroach infestations.

## 4. Discussion

The general public’s perception, attitude, and knowledge are important in the adoption of IPM in homes [16,30]. In the current study, the most commonly used materials for controlling cockroaches among those who reported pest presence were insecticide sprays, sticky traps, and baits; others included electronic repellers, cleaning, essential oils, and insect bombs. A similar survey conducted in the same community in 2015 (8 years prior to the current study) as a part of a community-wide IPM implementation study found 88% of those with pests (*n* = 50) used insecticides [19]. The top three used materials are sprays (83%), bait (7%), and dust (7%). In comparison, the current study showed a much lower percentage of residents using sprays and a higher percentage using sticky traps and baits. Both Raid and Hotshot spray and cockroach bait products were commonly found in retail stores in 2015 and 2023. There is no scientific evidence showing that electronic repellers [31,32,33], insect bombs [34,35], or essential oils [36] are effective in controlling field cockroach populations. Pyrethroid sprays are not very effective due to cockroach resistance [23,35]. Yet, the popularity of insecticide sprays used by residents indicates that periodic IPM education efforts are needed to reduce the use of ineffective insecticides and pest infestations. A survey of 648 residents from public housing indicated that residents spent 0.4–1.0% of their annual income on cockroach management, with 14% and 41% of them believing cleaning and applying insecticides were the best methods to control cockroaches, respectively [37]. Educating the public on IPM and the use of effective materials will reduce their financial burden from chronic pest infestations.

In this study, education did not lead to an improvement in the sanitation rating, which is consistent with a previous study where residents were offered a brochure and invited to attend a housekeeping class hosted by the community worker [22]. But education significantly improved sanitation ratings in apartments compared to the control in a study from Iran [38]. Another study showed sanitation level in the IPM-education phase (2.8) was significantly different from that in the Pre-IPM phase (3.9) and was accompanied by a decrease in trap catch; however, the study lacked a control group [39]. Therefore, the effect of education on improving house sanitation remains inconsistent. In terms of pest reduction, the effect of in-home IPM education plus provision of effective control materials resulted in better outcomes than those reported by McConnell et al. (2005) [26], which also provided both education and control materials. The 51% geometric mean count reduction in the control groups is likely due to a combination of residents’ self-administered control and monthly pest control services. However, the contractor was not aware of our study and did not change their routine service protocol during the study. Our findings are consistent with those reported by Jeong et al. (2006) [27] and Crider (2010) [28], which showed a significant reduction in cockroach counts or infestation rate. But one major difference from the above two studies is that our study also included offering cockroach control products to residents.

In the current study, we provided different cockroach control materials (gel bait and glue boards) than those used by McConnell et al. (2005) [26]. We did not know how much bait residents used since we did not find the bait tubes in subsequent surveys. Based on the measurement of boric acid dust bottles in 6 homes, residents used an average of 297 g of boric acid dust. The education part may have helped in enabling the residents to use the effective products on their own without relying on pest control staff. Whether offering effective control materials is the main factor causing cockroach reduction is unknown and needs to be studied. This can be determined by comparing education with education plus the provision of an effective pest management tool kit in the future. A similar study on cockroach allergen reduction found that education and application of gel baits by researchers resulted in 6 of 16 (38%) homes with trap counts reduced to zero, whereas only 1 of the 15 (7%) control homes had no trapped cockroaches after 6 months [40]. Besides the differences in product applicators, one major difference is that our study enrolled apartments with a minimum of 1 cockroach (4 traps/apartment, 7 days), whereas Arbes et al. (2003) study enrolled apartments with a minimum of 50 cockroaches (6 traps/apartment, 3 days) based on trap count [40]. Another study showed that applying highly effective cockroach bait alone by researchers resulted in a higher reduction in cockroach counts than the treatment by contracted professionals and the untreated control [41]. Both researchers and pest control staff-delivered bait applications resulted in significant cockroach reduction after 12 months, whereas the control homes did not show significant decreases in geometric mean numbers of trapped cockroaches except in the living room. This study indicates that the applicator’s knowledge and skills significantly influence cockroach control outcomes. Wang and Bennett (2006) [21] compared baiting alone with IPM (education, flushing and vacuuming, baiting) implemented by researchers in low-income households. After 7 months, the cockroach count reduced by 86% in the baiting alone treatment and 98% in the IPM treatment [21]. Both studies show that applying effective control materials alone can achieve high-level cockroach reduction and may account for the significant cockroach count reduction observed in the current study.

Most professional cockroach gel bait products are only available from online vendors, while boric acid dust and sticky traps are commonly found over the counter. The insect sticky traps and gel baits are more expensive than cockroach sprays. According to the Amazon.com website, the listed price for four tubes of Advion cockroach gel bait is $25.41 as of 7 April 2026. The price for Catchmaster Spider & Insect Glue Traps (45-pack, 15 sheets) is $19.99. The price for two cans (496 g each) of Raid Ant & Cockroach Killer Spray is $9.58. Raid Ant & Cockroach Killer Spray is the most affordable of the three products. However, over-the-counter pyrethroid sprays, including Raid Ant & Cockroach Spray, have poor residual efficacy against field-collected German cockroaches [18,23]. Harris Boric Acid Roach and Silverfish Killer Powder with lure costs $6.46 per bottle (453 g). It is a very affordable option and is an effective product when applied properly [42]. Providing residents with effective materials, along with instruction on proper use, could reduce residents’ complaints and requests for professional services, which is far more expensive than tenant-implemented control. Providing residents with glue boards as part of the intervention will help residents determine where to apply the cockroach control products and evaluate the success of their treatment.

Cockroach allergen levels are associated with cockroach density in homes [2]. A study in a high-rise building in New Jersey found that effective cockroach management reduced cockroach allergen levels (Bla g 1, Bla g 2) by >90% after 12 months [43]. This high allergen level reduction was accompanied by a very high (99.9%) reduction in cockroach counts. While significant, the current study shows cockroach counts reduced by only 56% after 6 months. Additional education and/or assistance from a professional pest control service may be necessary to achieve the desired results (e.g., full elimination) more quickly. A longer-term study (12 months) with multiple educational visits and provision of materials could also result in higher cockroach reduction. Challenges remain in changing people’s behavior and improving home sanitation levels, which are often associated with the abundance of cockroaches and other pests in low-income communities. Furthermore, many homes had structural defects allowing cockroaches to hide, move between apartments, or access water. These defects were not improved during/after our study period.

A limitation of this study is that the division of the two groups was not completely randomized, as more homes were assigned to the control group, and assignment to the treatment groups was partially based on the presence of residents at home. This could have biased the results. It is unclear whether those who were not at home during the day were equally receptive to adopting changes as those who received education and control materials. The in-house education format requires residents to be at home, which will be logistically inefficient and challenging to reach all residents in a building. An alternative approach is to notify residents to attend on-site seminars, which have their own challenges. Based on our on-site seminars in 16 communities managed by 4 housing agencies during 2023–2024 in New Jersey, the overall attendance rate was only 10% per community, even though free pest control materials were offered to residents, and in 14 of the 16 presentations, building managers provided free meals or refreshments. The on-site IPM seminars covered the prevention and control of household pests (cockroaches, mice, and bed bugs). Using a combination of on-site seminars, with built-in incentives to attend, plus door-to-door one-on-one education campaigns for residents who did not attend the seminar can reach more residents. Motivating and empowering the housing staff with knowledge of IPM may achieve more efficient delivery of IPM education and control materials. Studying the long-term effects of various approaches (education alone, education plus provision of pest control materials) on reducing pest control costs and pest infestation levels (cockroaches, bed bugs, mice) is needed. These studies will ultimately help design effective strategies to improve residents’ knowledge about IPM and self-implementation of effective pest control interventions.

## 5. Conclusions

Increasing public adoption of effective cockroach management practices is a challenging task. Key to our success in reducing cockroach infestations through resident-implemented control efforts lies in offering one-on-one brief education and providing effective, yet easy-to-use materials to residents. This format provides residents with the opportunity to interact with experts and have their specific questions about cockroach control answered. It is more effective to stimulate residents’ interest and make changes in their pest control practices, as shown by the significantly higher reduction in cockroach infestation levels. This case study verifies the value of this program in communities with frequent pest infestations. The lack of improvement in sanitation ratings indicates a limited effect of education on housekeeping behavior, and cockroach reduction was primarily due to the use of more effective materials. This approach generates cost savings by improving the efficacy of self-implemented control. The high percentage of residents using ineffective control methods at the beginning of the study, as revealed by this study and prior surveys, further underscores the need to educate residents in urban communities on the use of effective cockroach control materials.

## Figures and Tables

**Table 1:** Community characteristics based on the 26 residents who noticed the presence of pests in their homes at the beginning of the study.

Characteristics	*n*	Percentage
**Gender**	26	
Female		85%
Male		15%
**Race/Ethnicity**	25	
Hispanic		64%
African American		32%
White		4%
**Age group**	23	
Senior (>62 yrs old)		43%
Non-senior		57%
**Sanitation rating**	24	
1-Good		58%
2-Average		25%
3-Poor		13%
4-Very poor		4%
**Clutter rating**	24	
Few		71%
Moderate		8%
Cluttered		13%
Severely cluttered		8%
**Cockroach presence**	26	
Yes		58%
No		42%
**Mouse presence**	26	
Yes		58%
No		42%
**Bed bug presence**	26	
Yes		4%
No		96%

## Data Availability

The original contributions presented in this study are included in the article/Supplementary Materials. Further inquiries can be directed to the corresponding author.

## Supplementary Materials

The following supporting information can be downloaded at: https://www.mdpi.com/article/10.3390/insects17050483/s1. Supplementary Materials S1: Questionnaire; Supplementary Materials S2: Cockroach Prevention & Control.
