# Supplementary material for: Education and Provision of a Pest Management Tool Kit to Residents in Low-Income Housing: Effect on Cockroach Reduction
Source: Insects. 2026 May 9;17(5):483. doi: 10.3390/insects17050483 (PMC13207210; doi:10.3390/insects17050483)
Supplement: Supplementary file 1 [file insects-17-00483-s001.zip › insects-4103818-supplementary.pdf]

RUTGERS
School of Environmental
and Biological Sciences

Department of Entomology
Thompson Hall, Room 205
Rutgers, The State University of New Jersey
96 Lipman Drive
New Brunswick, NJ 08901-8524

www-rci.rutgers.edu/~insects/
changluw@rutgers.edu

848-932-9552
Fax: 732-932-9751

Supplementary material S1

Questionnaire

Date____________ Location: ________________________ Apt #_________

Senior/non-senior: _______Gender:_______ Ethnicity:___________________

1. How many years have you lived here? ______________

2. Do you see any of the following pests in your apartment?

Pest	Presence	If **NO**: When was the last time you saw these pests?	If **YES**: How often do you see them?	How long they have been present?	Do they bother you?
Cockroaches	Yes__<br>No __				Yes___<br>No ___
Bed bugs	Yes__<br>No __				Yes___<br>No ___
Rodents	Yes__<br>No __				Yes___<br>No ___
Other:<br>__________	Yes__<br>No __				Yes___<br>No ___

3. What you have done to control pests in the last 6 months? spray ___; dust ___; bait __; Other _____________

4. Are you satisfied with the pest control service offered by the building management?

   Yes_____ or No_______

RUTGERS | eIRB
APPROVED
IRB ID: Pro2021000491
Approval Date: 6/12/2021
Expiration Date:

RUTGERS–NEW BRUNSWICK
Entomology
School of Environmental and Bioloical Sciences

Supplementary material S2
Cockroach Prevention & Control

Why Cockroaches Are an Important Pest?

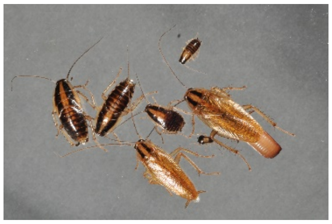

German cockroach

They Are a Serious Public Health Pest

- They contaminate food and surfaces (countertops, dishes, eating utensils, etc.).
- Their feces, skins, and dead bodies contain allergens which can cause asthma attacks.

They leave unsightly fecal spots

Prevention

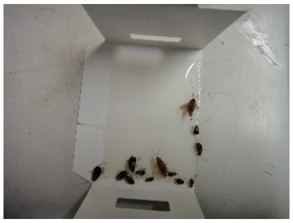

Glue board with cockroaches

Sanitation

- Clean up food residues and dirty dishes daily.
- Empty garbage can daily or seal the garbage can with tight lid.

Remove food and water sources

- Fix leaking pipes and remove empty water and beverage containers.
- Seal open food containers and pet food dishes.

Reduce their harborage sites

- Reduce clutter in kitchen and bathroom.
- Seal cracks, holes on the wall, pipes, and around cabinets.

Monitoring and Control

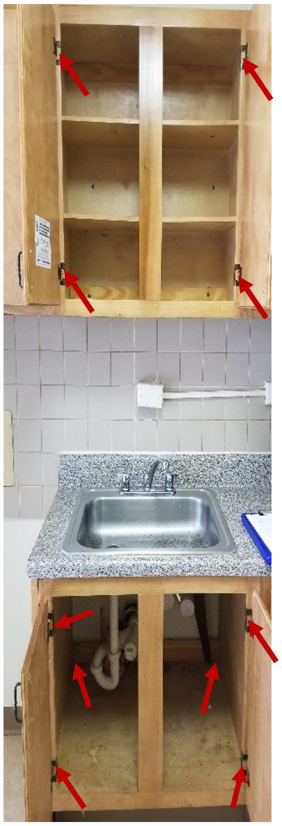

Arrows show where gel bait may be applied

- Place multiple glue board traps beside the refrigerator, stove, in the cabinets under the kitchen sink, beside toilet, at corners of the kitchen floor, or on kitchen counter.
- Use a vacuum machine to remove live and dead cockroaches. Remove vacuum bag, seal in a garbage bag and throw away.
- Apply small (1/4”) spots of cockroach gel bait in numerous spots in cabinetry, at corners and edges of appliances (stove, refrigerator). Apply a very thin layer of boric acid dust under and around appliances, along baseboards. Be sure to follow all label use directions on cockroach baits and boric acid products.
- Avoid using sprays or foggers. They are not effective and will contaminate the environment.
- Report to the management office or seek professional service if problem cannot be resolved soon.

For more information, contact Dr. Changlu Wang, Department of Entomology, Rutgers University, New Brunswick, NJ 08901. Phone: 848-932-9552. Email: Changluw@rutgers.edu
